# Supplementary figures and images for: Genome-wide identification, subcellular localization, and expression analysis of the phosphatidyl ethanolamine-binding protein family reveals the candidates involved in flowering and yield regulation of Tartary buckwheat (Fagopyrum tataricum)
Source: PeerJ. 2024 Mar 26;12:e17183. doi: 10.7717/peerj.17183 (PMC10979741; doi:10.7717/peerj.17183)

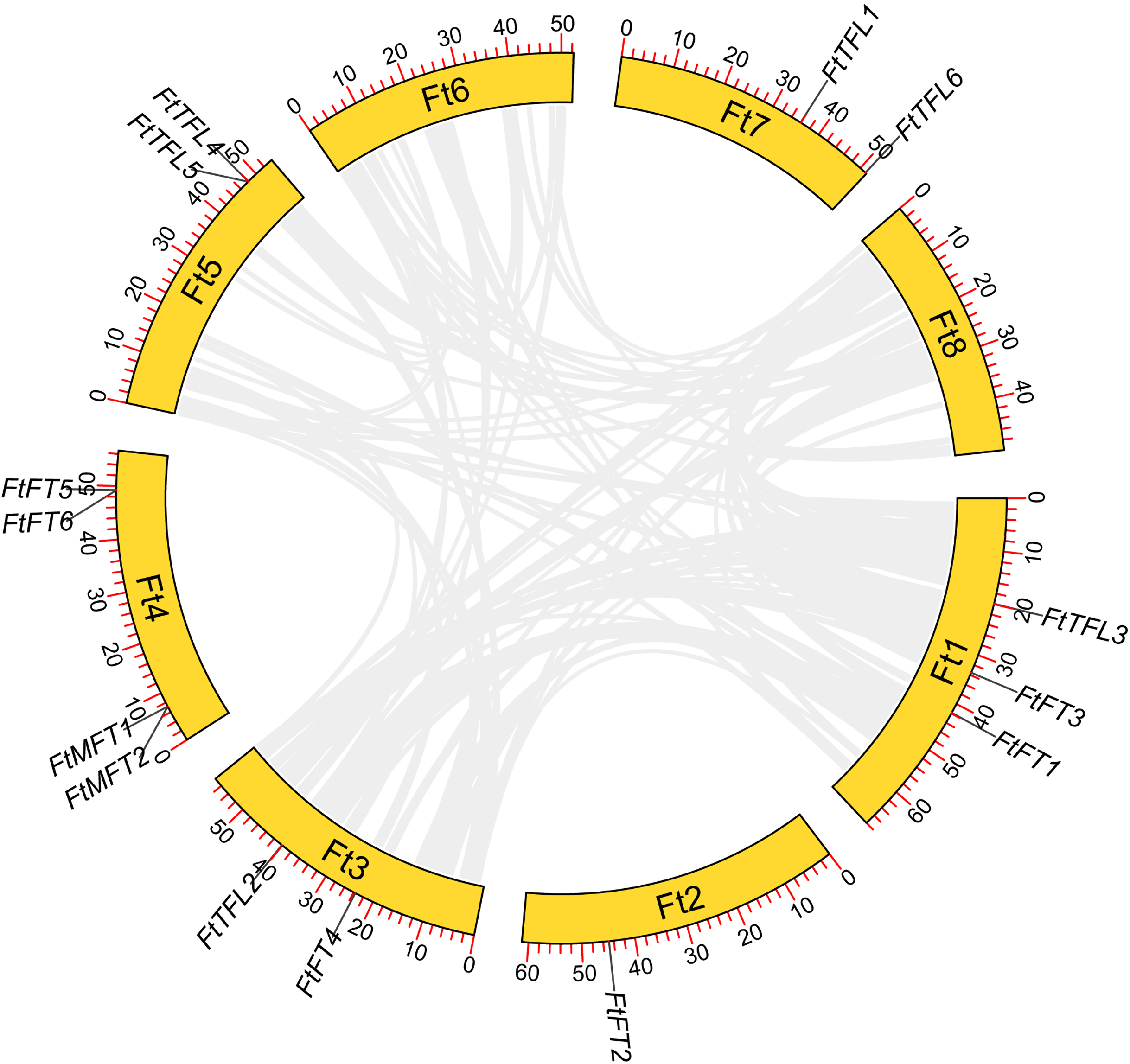

Supplement: Supplemental Information 1 [file peerj-12-17183-s001.png]

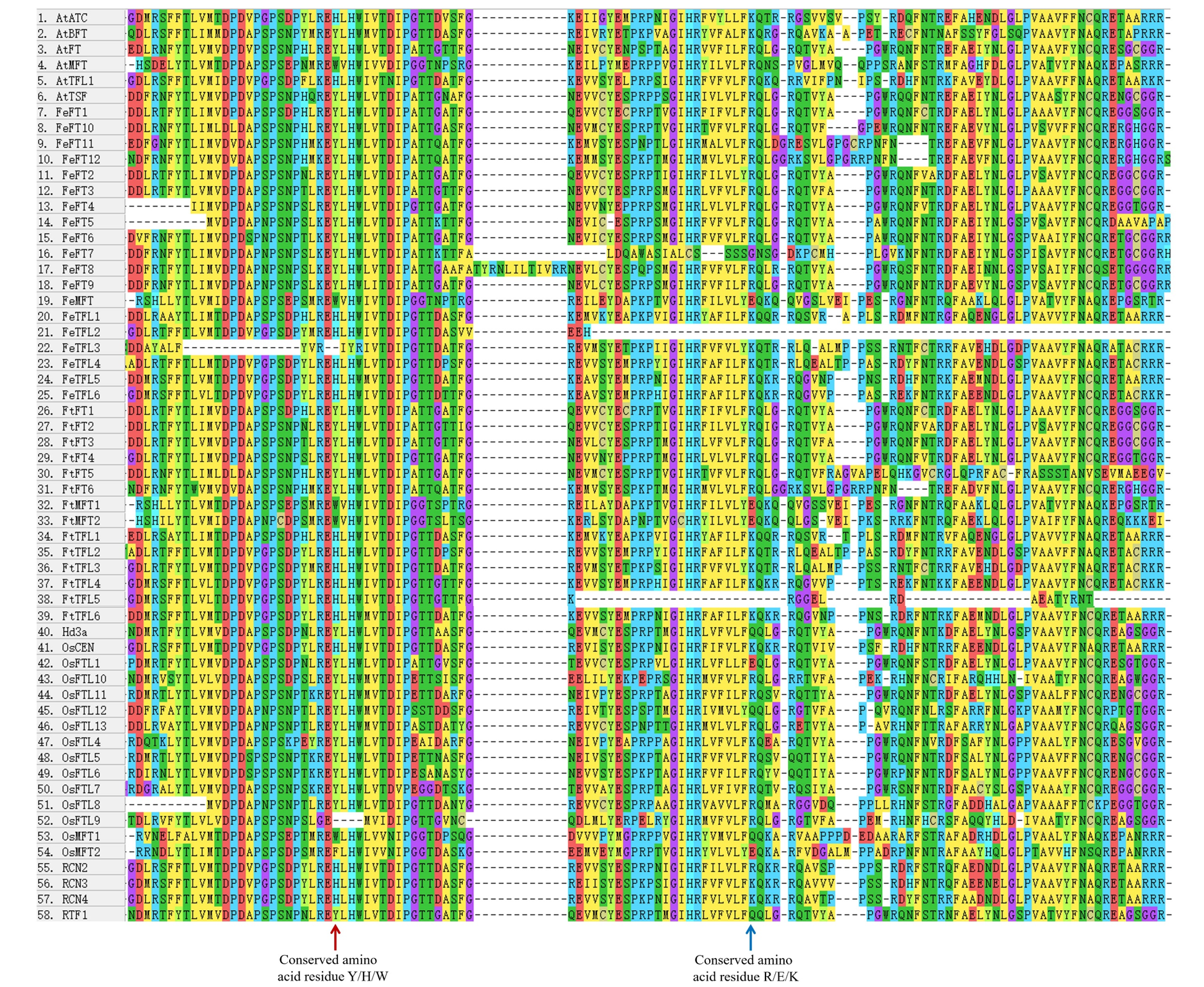

Supplement: Supplemental Information 2 — The red arrow indicated the key amino acids distinguishing FT-like (Y), TFL1-like (H), and MFT-like (W) functions. The blue arrow indicated the other key amino acids distinguishing FT-like (R), TFL1-like (K), and MFT-like (E) functions. [file peerj-12-17183-s002.png]
