# Supplementary material for: Genome-wide identification, subcellular localization, and expression analysis of the phosphatidyl ethanolamine-binding protein family reveals the candidates involved in flowering and yield regulation of Tartary buckwheat (Fagopyrum tataricum)
Source: PeerJ. 2024 Mar 26;12:e17183. doi: 10.7717/peerj.17183 (PMC10979741; doi:10.7717/peerj.17183)
Supplement: Supplemental Information 3 [file peerj-12-17183-s003.docx]

**Table S2** The qRT-PCR primers used in this study.

| **Gene name** | **Forward primer (5′→3′)** | **Reverse primer (5′→3′)** |
| --- | --- | --- |
| *FtFT1* | GGGCAAGAGGTGGTATGCTA | ACCAGGAGCGTATACCGTTT |
| *FtFT3* | TTATGAGAGCCCAAGGCCAA | AGGCAAACCGAGGTTGTAGA |
| *FtFT4* | GGAACAACCGGAGCAACTTT | CCGGCAAACCGAGGTTATAG |
| *FtFT7* | AGCCATGGTCACAGGATTGA | GATGGCTACGAGAACTGCCT |
| *FtTFL1* | AAGAGTGGTGGGAGAAGTGG | GAGAGCAAGAGAGGAGGCAT |
| *FtH3* | GAAATTCGCAAGTACCAGAAGAG | CCAACAAGGTATGCCTCAGC |
